# Changes in the Association between European Workers’ Employment Conditions and Employee Well-Being in 2005, 2010 and 2015

**DOI:** 10.3390/ijerph17031048

**Published:** 2020-02-07

**Authors:** Juan A. Marin-Garcia, Tomas Bonavia, Josep-Maria Losilla

**Affiliations:** 1 Department of Organización de Empresas (Business and Management), Universitat Politécnica de Valéncia, Camino de vera s/n, 46022 Valencia, Spain; 2 Department of Social Psychology, University of Valencia, Av. Blasco Ibáñez, 21, 46010 Valencia, Spain; 3 Department of Psychobiology and Methodology of Health Sciences, Autonomous University of Barcelona, carrer Fortuna Edifici B, 08193 Bellaterra, Spain

**Keywords:** employment conditions, anxiety, fatigue, dissatisfaction, european workers, labor conditions, workforce, workplace wellness, mental health

## Abstract

The aim of this paper is to study whether there is a change in the association between employment conditions and European employees’ well-being at three different time points (the years 2005, 2010 and 2015), characterized by different socio-economic contexts. We based our study on the European Working Conditions Survey. Logistic regressions were performed by adjusting for gender, age, level of education, seniority, occupation, establishment size, activity sector and economic activity. Adjusted odds ratios (ORadj) and 95% confidence intervals (95% CI) are reported. In general, the association between employment conditions (type of employment contract, supervising, weekly working hours, long working hours, other paid jobs, working at weekends or doing shifts) and well-being indicators (anxiety, fatigue and dissatisfaction) seemed to continue being harmful, or had even changed for the worse since 2005. The paper briefly discusses the possible reasons for this situation and calls for future research on the relation between well-being and irregular type of contracts, self-employment, supervising others or hours worked per week. Some implications in public health policies are also discussed.

## 1. Introduction

Labor conditions (employment conditions, working conditions, employment relations, risk factors, psychosocial work factors…) that affect workers’ well-being have been extensively studied, for example, in the different reports by the European Agency for Safety and Health at Work (EU-OSHA) [1], and other studies [2,3,4,5,6]. There is less research about changes in these conditions over time and their associations with employees’ mental health and satisfaction [7], and even less about whether the relationships of these conditions on workers’ well-being remain the same over time [6,8,9,10,11]. Considering this lack of investigation, we propose to find out whether there was a change in the relationship between employment conditions and well-being indicators (anxiety, overall fatigue or dissatisfaction) on European workers between 2005, 2010 and 2015.

Some well-known studies have not focused specifically on analysing the changes that have occurred, as they only mention them [12,13,14]. Other studies have focused on specific working populations and activity sectors, or on only one country [2,5,11,15,16,17,18,19,20,21,22], or on very few countries in the same geographical area [23,24], reducing the possibility of generalizing the results. Other studies have concentrated on analysing the changes in some type of variables (for example, psychosocial work factors), or the evolution of employment conditions over time, but without examining the associations of these changes on employees’ well-being [7,25]. Other studies that have related different aspects of labor conditions to well-being have not analysed these relationships over time or the results specifically produced by employment conditions [6,26,27,28,29,30].

From the few studies carried out in Europe to find out whether the repercussions of employment conditions can vary over time, the following ideas stand out. Benach et al. [8] reached the conclusion that the prevalence of all the physical and mental well-being indicators in 2000 showed a slight increase; that is, they were worse than in 1995. In general, distribution of health indicators by type of employment was very similar in both surveys. In their final report, Welz et al. [31] corroborate that, with very few exceptions, in Europe since the crisis, countries saw a drop in absenteeism and an increase in presenteeism, along with a rise in job satisfaction. However, Lopes et al. [32] show that there has been a significant reduction in the percentage of satisfied workers during the period from 1995 to 2010. Parent-Thirion et al. [14], meanwhile, point out that the high level of satisfaction has remained stable or, in any case, only declined slightly during 2000–2010 decade, with the economic downturn after 2007–2008 not appearing to have affected employee satisfaction. Some studies [31,33,34], although not all [8,32], analyse the variables independently and, when proposing relationships between variables and changes over time, use only descriptive data to separately analyse each variable, showing that there seem to be common tendencies. Therefore, none of the conclusions drawn from these studies has been adjusted for a set of variables that the literature has established as having a differential result in workers’ well-being (such as sociodemographic variables, occupation, activity sector, and economic activity).

In addition, to date, more attention has been paid to the prevention of psychosocial risks [1,35,36] than to the possible effect of changing employment conditions on well-being. The challenge we propose in this article is to fill this gap by using harmonized European data in such a way that it is possible to draw general conclusions for all types of occupations, activity sectors, and economic activities. For this purpose, we use the European Working Conditions Survey (EWCS) in three periods with very different socioeconomic contexts (2005, 2010, and 2015). One of the overall aims of the EWCS is to measure labor conditions across European countries and monitor trends over time. The EWCS is highly valued by policymakers and researchers as an important and distinctive source of comparative and in-depth information [36].

We chose the years 2005 and 2010 because it is well-known that, in Europe especially, there was a severe economic crisis that had effects on the employment conditions of its citizens [37,38]. We also add the last available European Working Conditions Survey (EWCS6) with data form 2015 [39], to check the trends several years after the crisis and analyse pre- and post-crisis years [5]. It is evident that the changes we might show cannot be blamed only on the effects of the crisis. They also reflect developments that occurred before the crisis [40,41] due to the phenomenon of globalization, the search for greater flexibility at every level (decentralisation of collective bargaining, intensification of work…), and the tertiarization process [9].

However, the threat to the workers well-being posed by the economic crisis of 2007–2008 is widely recognized in the scientific literature, particularly in the context of the EU, among other things because quality jobs are replaced by jobs with lower wages and worse working conditions, accentuating the increase in flexible and precarious employment arrangements, and increasing job insecurity [10,12,25,31,42].

Knowing the potential changing associations between employment conditions and workers’ well-being makes it possible to better adapt public policies, focusing first on improving the employment conditions that have larger and more stable relationships with labor well-being. In addition, those employment conditions whose relationship with on well-being vary more for circumstantial reasons [43,44,45] should be taken into account by these programmes in order to make the appropriate adaptations when these variations are expected to occur (for example, during periods of economic recession). In summary, as Morley [36] points out, if interventions are to be effective, public policies need to take into account the links and interactions between what goes on in the workplace and what is happening in society in general. According to the aforementioned, this study analyses whether the change in the socioeconomic context is related to a change in the association between the employment conditions and well-being of European workers.

## 2. Materials and Methods 

The EWCS is conducted by the European Foundation for the Improvement of Living and Working Conditions (Eurofound), which is an autonomous EU agency, funded by the general budget of the European Commission. The EWCS series began in 1990–1991, and it is generally conducted once every five years. The survey is based on a questionnaire administered face-to-face to a random sample of ’persons in employment’ (i.e., employees and the self-employed), representative of the working population in each EU country. When a suitable sampling frame (register) with addresses/persons is not available for a country, the random route method is used to select the households and individuals. No quotas or similar non-random solutions were implemented [14,46,47,48].

The EWCS coverage over the years reflects the evolution of the EU, as more states have joined over time. In 2005, the survey included 31 countries, including the 27 current EU member states (EU-27) at the time. In 2010, the fifth wave covered 34 states. The sixth wave of the survey (2015) was the most comprehensive so far in terms of geographical coverage; it covered the EU-27 Member States and eight other European countries [39]. In this paper, we analyse the EU-27 data.

The statistical population includes all persons aged 15 or over who are in employment during the reference period. A person is considered in employment if he or she did any work for pay or profit during the reference week for at least one hour. This is the same definition as the one used in the Labor Force Survey [49], and the same inclusion and exclusion rules apply. In most countries, the target number of interviews was between 1000 and 2000, with the exception of smaller countries (Estonia, Cyprus, Slovenia, Malta, and Luxembourg), where the target was 600 interviews in 2005. In all, the EWCS aimed to complete 29766 interviews in 2005; 42900 in 2010 and 43000 in 2015. In our case, the sample for the EU-27, the setting for this study, consisted of 24526 employees (55.8% male; 44.2% female) in 2005; 33190 employees (55.1% male; 44.9% female) in 2010 and 32738 employees (52.4% male; 47.6% female) in 2015.

The aim of the EWCS is to provide an overview of the state of labor conditions –understood in a broad sense- in the EU, in order to identify major issues and changes affecting the workplace and contribute to better monitoring of the quality of work and employment in Europe. The number of questions and issues covered in the EWCS has expanded in each subsequent survey, but a core set of key questions has remained unchanged to facilitate the study of trends in employment and working conditions. The EWCS questionnaire includes more than 100 items on a wide range of issues related to the employment conditions and related variables [46]. The questionnaire was created by Eurofound and tested in various ways to ensure that it provides a valid measurement of the concepts surveyed. 

Uniformity during data collection was ensured through the development of guidelines and training material for all respondents, subsequently translated into all the national languages (for a more detailed description see [47]). These data provide a unique and comparable source of information about the working conditions in different European countries (the same questionnaire was used in all the countries covered). The data from the 2005 and 2015 waves of the EWCS included in the Integrated Data File (under SN 7363 http://doi.org/10.5255/UKDA-SN-7363-7) were used for the analysis [50].

The definitions of the variables in this study were based on the report by Parent-Thirion et al. [14] (see Appendix C for definition of variables and descriptive statistics). Below, we describe the transformations made (SPSS syntax details for variables’ transformation are provided in Appendix A). 

*Well-being*. Our response variables related to workers’ well-being can be grouped in two types. (a) In the first type of variables, respondents were asked to indicate whether they had suffered from anxiety (depression or anxiety in 2005 and 2010), or overall fatigue in the past 12 months (yes/no). (b) The second type of response refers to satisfaction, measured by the following question: “On the whole, are you very satisfied, satisfied, not very satisfied, or not at all satisfied with the working conditions in your main paid job?”. The use of single-item scales for well-being variables is considered a valid procedure [51,52]. We recoded the responses as a dichotomous variable (satisfied/not satisfied) to obtain enough cases in the crossings among the various exposure/fit variables levels to facilitate the interpretation of the results as other research works have done [1,15,53,54,55]. 

*Employment conditions*. The employment condition measures are our exposure variables. We are going to focus our research on the analysis of the employment conditions by distinguishing them from other types of variables such as working conditions or employment relations [5,37,56]. Benach et al. [25] and Van Aerden et al. [57] clarify that employment conditions have to do with the mutual agreements between employees and their employers about the employment organisation in terms of contract, working hours, etc. However, working conditions refer to the general physical, ergonomic, biological, chemical, and psychosocial environment at work and various risks. Employment or industrial relations refer to the way all stakeholders at work interact with each other, both in a formal (such as collective bargaining processes) and informal (such as contact with their supervisor or social support) sense. Specifically, we are going to address the following nine employment conditions: (a) Type of employment contract and self-employment, with 4 categories: permanent contract, temporary contract, self-employed, and no contract & others; (b) supervision, with two categories: with no subordinates and with one or more subordinates; (c) weekly working hours: less than 20 h, between 20 h and 35 h, between 36 h and 45 h, and more than 45 h per week; (d) long working hours, defined as: working more than 45 h per week and more than 10 h per day at least once a month (two categories: yes/no); (e) atypical working days to distinguish those who work weekends from the rest (yes/no); (f) shift work, with two categories (yes/no); and (g) alternating/rotating shifts to differentiate those who work set shifts from those who work rotating shifts; (h) other paid jobs (yes/no).

*Year of survey*. To meet the main objective of our research we used the year of survey (2005, 2010 or 2015) as moderator of the relationship between employment conditions and well-being indicators. 2005 represents the pre-crisis scenario. Between 2007–2010 there was a very deep economic crisis in Europe that affected all countries (although not in the same way), and it was considered away in 2015 as evidenced by numerous studies [12,14,25,31,32,33,42].

*Control variables*. All of our results have been adjusted, taking into account a set of variables established in the literature that can differentially influence the relationship between the employment conditions and workers’ well-being [12,13,14,25,31]. These potential confounding or control variables are the following: sex (male/female), age (16–24; 25–34; 35–54; 55 or more), education level (pre-primary or primary; lower, upper and post-secondary; tertiary education, first and second), seniority (1 year or less; more than 1 to 5 years; more than 5 years), establishment size (number of workers in the workplace: 1–9; 10–249; 250 and over), activity sector (public; private; other), economic activity (industry; services; public administration and defense), and occupation as main paid job (isco9—Elementary occupations; isco8—Plant and machine operators and assemblers; isco7—Craft and related trade workers; isco5—Service workers and shop and market sales workers; isco4—Clerks; isco3—Technicians and associate professionals; isco2—Professionals; isco1—Legislators, senior officials and managers. The missing categories were eliminated because of an insufficient number of cases, which made it impossible to perform the statistical analyses). 

Logistic regressions were performed to investigate the association between the workers’ employment conditions and well-being, adjusting for relevant characteristics of respondents and their workplaces, as described above. Separate logistic regression models were adjusted for each employment condition due to the non-convergence of the estimation algorithm by including all exposures and adjustment variables together in a single regression model [15,44] Although this practical approach does not allow the analysis of the possible correlation between exposures, due to random selection of all EWCS samples, the estimated effects can be considered highly representative of the natural groups independently characterized by all levels of the exposure variables investigated. Furthermore, we tested the interaction between the employment conditions and the year of the survey to assess whether the relationship between the employment conditions and well-being was the same in 2005 and 2010, and in 2010 and 2015. 

As data were obtained from probabilistic samples at all the three studied time points, the three EWCS data series jointly make up a longitudinal populations design. The statistical validity of the comparison made among these series lies in the high representativeness and the maintenance of the same procedure and questionnaire to collect the data in each series, as well as including in the regression models both the main adjustment variables and the effect of the interaction among the analyzed exposure variables and the time when data were collected. This allows an analogous interpretation of the results with which a longitudinal panel design was made.

Logistic regression models were fitted using IBM SPSS Statistics v22.0 package (SPSS syntax details for logistic regresdions are provided in Appendix B). The results of the association between employment conditions and well-being are presented as adjusted odds ratios (ORadj) and 95% confidence intervals (95% CI). Cross-national weights necessary to produce European figures were computed by multiplying each national weight by the country’s proportion in the EU-27 region, and statistical analyses were conducted by weighting the relative size of the workforce in each of the European countries.

## 3. Results 

### 3.1. Relation between Well-being and Employment Contract

Regardless of the year (see Table 1), and based on the significance of the results, holding a temporary contract did not differ much for anxiety compared to having an indefinite contract. Nonetheless, having a temporary contract involved an increase in the odds for fatigue and dissatisfaction *versus* holding an indefinite contract.

Being self-employed was associated with increased odds of having anxiety or fatigue compared to holding an indefinite contract for all the three study years, but was related to a slight drop in the odds of dissatisfaction in 2005 and 2010, while more dissatisfaction clearly came over than for the permanently employed people in 2015. 

The association between people who worked with temporary contracts or being self-employed and well-being did not change from 2005 to 2010, and no changes were observed between 2010 and 2015 (save dissatisfaction as mentioned above, which was more negatively marked for the self-employed).

The odds of experiencing anxiety, fatigue or dissatisfaction in the people with no work contracts varied for all three studied years. In 2005, compared to employees with a permanent contract, the workers with no contract reported less anxiety, fatigue was statistically the same and dissatisfaction was slightly higher. In 2010, these three problems were significantly marked for this group. Nevertheless, in 2015, anxiety was no different for the workers with no contract and those with indefinite contracts, although differences were found in 2005 (less anxiety in the workers with no contract) and 2010 (more anxiety in the workers with no contract). After the increase in 2010, fatigue in 2015 once again did not differ among the workers with no contracts and those with indefinite contracts, which occurred in 2005. Dissatisfaction, which was higher in 2010 than in 2005, significantly rose again in 2015 compared to 2010.

### 3.2. Relation between Well-Being and Supervising

The relation between supervising other people and anxiety or fatigue has grown over the years, although significant differences were seen only when comparing anxiety between 2010 or 2015 with 2005, or when comparing fatigue in 2015 with 2010 or 2005 (Table 2). In 2005, very little difference was observed between being a supervisor or not, and anxiety or fatigue was less for people who supervised others. However, the anxiety or fatigue associated with supervising others became worse in each dataset. The data clearly revealed an association with being a supervisor and less job dissatisfaction, which was weaker (a significant change) between 2010 and 2015.

### 3.3. Relation between Well-Being and Long Working Hours

Long working hours were related to worse results in all the considered response variables (see Table 2). This relation remained stable when comparing all three studied years. The effects were stronger on fatigue and anxiety than on dissatisfaction for all the considered years. In other words, it would seem that a long working day (working more than 10 h a day several days a month) was related to worse well-being compared to those employees who did not have such long work schedules. The odds for anxiety and fatigue were more than double, and came close to being 50% higher for dissatisfaction.

### 3.4. Relation between Well-Being and having Another Paid Job

Having another paid job, either occasionally or regularly, was not so strongly associated with anxiety in 2005 and 2015 (a 20% increase in the odds compared to having only one job). This association disappeared in 2010, and no differences were observed between the people with one job and those with several. Not a very marked tendency appeared to reduce the relation between having another paid job and more fatigue. Indeed, in 2015 no effects were found, but in 2005 the people who did several jobs reported more fatigue than those with only one job. Job dissatisfaction was higher (45% higher in 2005 and approx. 20% higher in 2010 or 2015) among those workers who had several paid jobs. The changes in the intensity of this association were not significant among the three studied years.

### 3.5. Relation between Well-being and the Number of Weekly Working Hours

Regarding the number of weekly working hours (Table 3), working 45 h or more per week was clearly associated with worse well-being, regardless of the year and for all the considered variables. No marked differences appeared for working between 20 and 35 h/week with the reference group, but belonging to this category led to a neutral association, or even to less anxiety, fatigue or dissatisfaction in 2005 by being significantly related to higher anxiety, more fatigue and greater dissatisfaction in 2010 or 2015. Compared to the people who worked between 36 h and less than 45 h, having shorter work weeks (<20 h) appeared to be generally associated with better well-being in all the studied years. However, this beneficial effect seemed to weaken with time, and a clear change in anxiety and dissatisfaction appeared between 2005 and 2015. For fatigue, the change in the association appeared in 2010.

### 3.6. Relation between Well-Being and Atypical Working Days, Shift Work andAalternating/RotatingSshifts

Working at weekends (atypical working days), work shifts, and working alternating or rotating shifts, were associated with higher anxiety, fatigue and dissatisfaction (Table 4). For anxiety, working on atypical working days was associated more than working set shifts or alternating/rotating shifts. Working set shifts led to an increased association with anxiety from 2005 to 2010, which lowered in 2015 to levels that were similar to, or lower than, those in 2005. Alternating/rotating shifts modestly increased the odds for anxiety (about 20%), and they remained stable throughout the three time points. Similarly, fatigue was more associated with working on atypical days. In 2015, the negative association once again increased (the odds of evidencing fatigue from working on atypical working days almost doubled that for working only Monday-Friday). When we analyzed dissatisfaction, these three employment conditions presented very similar increased odds (an increase of around 40–60%).

## 4. Discussion

The relation between employee conditions and the three well-being variables remained with no major changes taking place over time for the employees with temporary contracts, long working hours, with other paid jobs, working on atypical working days or working any kind of shifts. In nearly all these circumstances, the odds of suffering anxiety or fatigue or dissatisfaction increased compared to the reference category for each employment condition.

Between 2005 and 2010, Europe went through a severe economic crisis. No one seems to question that this crisis produced a worsening in employment and job conditions [16,17,23,32,37,40]. Curtarelli et al. [33] indicate that the crisis may have influenced workers’ subjective perception of their work situation and its effects on their physical and mental well-being. Some studies posit that workers’ perceptions of the work environment are likely to be derived not only from the personal and organizational context, but also from the external macroeconomic context [16,58]. As an example, Welz et al. [31] indicate that the economic crisis has caused an increase in job insecurity across Europe, along with specific changes in labour regulations. As a result, employees have growing concerns about having or keeping their jobs and maintaining their incomes, which affects their well-being and health [12]. Findings from the report on the impact of the crisis [42] show evidence of an increase in some psychosocial risks linked to changes that took place during the economic crisis (such as higher unemployment, more flexible labour regulations, and restructuring processes), which have had a negative effect on workers’ well-being [10]. Therefore, we could expect a change for the worse in well-being. One the one hand, the association between worse well-being and certain employment conditions which became more marked after the crisis remained (temporary contracts, long working hours, working shifts or weekends) [29,38,59,60,61,62].

On the other hand, we found that the negative effect on anxiety, fatigue and dissatisfaction were intensified for some employment conditions. For instance, for those workers with no contract, who in 2005 had 31% less odds of suffering anxiety than those with indefinite contracts had 30% higher odds in 2010 (a change of around 60% took place in the odds during this period). Likewise in 2005, the odds of presenting fatigue did not differ between not having a contract and holding an indefinite contract. In 2010 however, the odds had increased by 43%, and then lowered in 2015 before returning to the level they were at in 2005. As for both these outcomes, in 2005 the association was the opposite to what was expected. Worse employment conditions (no contract) were linked with less anxiety and fatigue than having an indefinite contract. Yet in 2010 (halfway through the crisis), the association reversed and was linked with more anxiety and fatigue. In 2015, the situation became more like that of 2005. That is to say, during economically good periods (2005 and 2015), not having a contract did not imply suffering more anxiety or fatigue than having an indefinite contract [63], but the likelihood of suffering anxiety or fatigue sharply rose during the crisis. However, further future research will be necessary to explain this tentative proposal because the available literature about it is scarce [30] and, according to the study sample, dissatisfaction was greater for those workers with no contracts than it was for those with indefinite contracts in all three studied years. Moreover, the association with dissatisfaction significantly increased in 2015 (with a 73% increase in the odds). 

We also found a closer association between dissatisfaction and self-employment. Despite self-employment being associated with suffering more anxiety and fatigue than having an indefinite contract, in 2005 and 2010 it was linked with lower odds for dissatisfaction than for holding an indefinite contract. In 2015 it negatively jumped by 39% and self-employment was associated with a 21% increase in the odds for dissatisfaction. In 2015, the three well-being measures became more negative in self-employment situations than for the workers with indefinite contracts. This situation could perhaps reflect both the positive aspects of self-employment in certain groups before the crisis [64] and its effects [65] and the complexity of different types of self-employment (e.g., voluntary versus forced) and the outcomes deriving from them [66,67]. We believe that studying well-being and self-employment is an emerging theme and further future research will be necessary to analyze this association [68].

Changes also appeared in the effect of supervising others on well-being, which caused more anxiety and fatigue in 2010 and 2015, when it was linked with 13–11% lower odds of suffering these outcomes in 2005. Although it still appeared as a source of protection against dissatisfaction, its effect on the odds went from being about 40% lower in 2005–2010 to only 24% lower in 2015, and could be linked with the growing anxiety and fatigue trend, Moreover, perhaps working as a supervisor in an organizational context has become more stressful after the crisis. Nevertheless, very little specific research has been conducted between well-being and having jobs that involve supervising others. Therefore, it would be interesting to open future research lines on this matter.

We also stress the relation that has become well-established between hours worked per week and effects on well-being [69]. In 2005 and 2010, the fewer the hours worked per week, the greater well-being was. In 2015 however, working between 20 and 35 h/week or more than 45 h/week was associated with worse well-being than for the 36–45 h/week schedule, which was not that different to working fewer than 20 h/week. Working fewer than 20 h/week led to lower odds for anxiety, fatigue and dissatisfaction. So it would appear to be a positive condition for well-being when compared with working between 36–45 h/week, perhaps due to the conciliation possibilities that it creates for workers [38,60]. However, this effect has become weaker with time and has gone from 40–50% odds of suffering fewer negative effects to become almost equal to working 36–45 h/week. Maybe the possible advantages of conciliation and having free time with this contract type fade when combining it with the inconveniences of earning less income, having few stable job perspectives or leaving a series of mini-jobs behind without the possibility of improving one’s professional promotion [38,69]. Likewise, working between 20 and 35 h/week was not related to worse well-being than working normal hours (36–45 h.) in 2005. After 2010, the odds of suffering anxiety, fatigue or dissatisfaction were higher than the reference category. We believe that the causes underlying this trend could be the same as for working fewer than 20 h/week. Finally, we stress that between 2010 and 2015, the odds of suffering anxiety when working more than 45 h/week sharply rose and became 126% higher than for those working normal working hours, although these relations could be conditioned by specific working conditions [34,70,71].

If we now broaden the focus of the policies, Benach et al. [25] argue that policies that impose more flexibility on the European labour force should also take into account the possible effects on the well-being of the employees affected. According to the European employment policy, high employment rates and high-quality jobs are not mutually exclusive. Instead, good-quality jobs are an important precondition for improving occupational well-being [2,25].

There is a danger that, organizations might be tempted to focus on survival (through changes in organizational policies such as temporary contracts, job losses or atypical working times) rather than improving the psychosocial work environment. Cost-cutting measures that might seem logical in the short term could, however, cause serious medium- and long-term consequences for people’s well-being, both individually and collectively [10]. Therefore, our results help to highlight the need for a focus on employment conditions as a way to better understand the changes in workers’ well-being, even in times of deep crisis.

From a more general point of view, public policies, both social and employment-related, should pay greater attention to the pursuit of the long-term strategic goal of promoting a generalized improvement in living and labor conditions [36,43,44,45]. In fact, various results stemming from the different EWCS have been included and developed in different European policies [36,39]. Examining the way employment conditions have changed (or remained the same), and their association with workers’ well-being can shed light on the progress being made in moving toward a more integrated, competitive, and sustainable Europe [40].

It is necessary to make some observations about our study. Our results are not based on a longitudinal design, but rather on the analysis of three comparable cross-sectional random samples. The inability to empirically rule out the possibility of reverse causation is a common limitation of cross-sectional studies in occupational stress research and epidemiology [25]. For this reason, a longitudinal perspective is extremely valuable in controlling effects on workers’ well-being. In any case, there is evidence that work and employment seem to be causally related to well-being, rather than the contrary [11,25]. In addition, indicators of well-being in the different EWCS are self-reported by the respondent, which has its limitations. Likewise, the measurement of well-being indicators with simple and crude indicators may have limited the possibility of detecting significant associations [8]. However, most of the indicators on self-reported well-being are commonly used in international studies, and many of them have been validated in previous research [1,51,52]. A correlation between the employed independent variables could also exist. We herein opted to analyze and report the results of each one separately. It would be convenient for future research to design alternatives to examine them in groups.

It is certain that the general view of relations sketched here at a global level for the EU-27 could be different for each country at the time points considered [9]. For example, the crisis in Europe has had a differentiated impact on working conditions [31] and psychosocial work factors [7]. We think that the control variables considered in this study make it possible to more accurately establish the relationship between employment conditions and well-being, as these conditions can vary among countries and within the same country. Our results can be generalized to any similar group of workers without considering their country of origin, thanks to the control of some key variables. Welz et al. [31], among others, also recognize the importance of these adjustment variables. Furthermore, other studies have not found clear differences among countries after eliminating the effects of certain confounding variables. For example, Benavides et al. [41] and Benach et al. [8] found that patterns were generally consistent across countries, and associations between employment categories and physical and mental well-being indicators remained virtually identical after country level variables were included in the models. Ardito et al. [12] found that well-being and safety at work depend on certain work variables that, when taken into account, cause cross-country differences to tend to disappear. Malard et al. [6] state that “for the large majority of the countries, the changes were not different from the mean European changes for all factors” (p. 1137). According to Morley [36], although major differences remain among countries, the evidence indicates that changes in the European policy environment are taking place as the EU evolves and integrates. Much of the traditional intra-EU stereotyping of countries in terms of their socioeconomic systems is increasingly misplaced. Elements of different labour market and welfare policy incentives and support arrangements are crossing boundaries. In fact, the country variable is only an aggregate of many other variables (not only labour, but also social, cultural, environmental…) that undoubtedly should be considered when designing national policies, but are less relevant when trying to design a policy framework for all of Europe. It seems that, as Parent-Thirion et al. [14] state, looking at changes in work over time, as measured by the EWCS series, shows limited changes globally, and the overall results do not fully reflect the emphasis in European policymaking during the past 20 years. There remains, therefore, a long way to go.

## 5. Conclusions

Europe suffered a severe economic crisis between 2005 and 2010, which it had recovered from in 2015. Our study, based on three comparable cross-sectional surveys, examined whether employment conditions were related to workers’ well-being in the same way at three different time points, without this result being confounded by the influence of other variables that the literature has established as possibly intervening in these relations. 

In general terms, and with the same situation for the adjustment variables (gender, age, level of education, seniority, occupation, establishment size, activity sector and economic activity), the people with indefinite contracts, no supervising responsibilities, who work between 36 and less than 45 h/week, do not work long working hours, do not have several jobs, do not work shifts (neither set nor alternating/rotating shifts) and do not work weekends report less anxiety, fatigue or job dissatisfaction than those in any of the other categories of each employment condition herein analyzed. That is, worse employment conditions lead to higher odds of suffering anxiety, fatigue or dissatisfaction compared to the reference category. Nonetheless, we observed that this relation did not always appear in this way for all the studied years for some working conditions (employment contracts other than indefinite contracts, supervising others and weekly working hours). 

We believe that, although the relationships between work and well-being are complex and probably do not materialize in only one direction, work can have positive consequences for individuals’ well-being [1], but can also have negative consequences if employment conditions are unfavorable. In fact according to the analyzed data, the relation between employment conditions and employee well-being seemed to remain or increased in intensity for the years between 2005 and 2015. Worse employment conditions were associated with less well-being and socio-economic conditions, or this effect simply increased over time for some conditions, while intensity remained for the rest. 

## Figures and Tables

**Table 1:** Relationship between well-being and employment contract in 2005–2015 (reference: indefinite contract).

		No Contract		Self-Employed		Temporary	
DepVariable	Year	OR_adj_ 95% CI	BY	OR_adj_ 95% CI	BY	OR_adj_ 95% CI	BY
Anxiety	2005	0.69 (0.52;0.90) *		1.22 (1.04;1.43) *		0.80 (0.66;0.98) *	
	2010	1.30 (1.00;1.68) *	↑	1.45 (1.23;1.71) *		1.00 (0.82;1.22)	
	2015	1.10 (0.87;1.39)		1.51 (1.32;1.72) *		1.06 (0.91;1.22)	
Fatigue	2005	0.86 (0.74;1.01)		1.38 (1.24;1.53) *		1.32 (1.18;1.48) *	
	2010	1.43 (1.22;1.67) *	↑	1.33 (1.20;1.48) *		1.37 (1.22;1.53) *	
	2015	0.91 (0.75;1.09)	↓	1.29 (1.17;1.42) *		1.28 (1.15;1.42) *	
Dissatisfacion	2005	1.19 (1.02;1.38) *		0.77 (0.68;0.88) *		1.51 (1.35;1.69) *	
	2010	1.32 (1.13;1.54) *		0.82 (0.72;0.93) *		1.26 (1.13;1.41) *	
	2015	2.05 (1.76;2.40) *	↑	1.21 (1.08;1.36) *	↑	1.54 (1.39;1.71) *	

N weighted 67993 (anxiety); 68002 (fatigue); 69158 (dissatisfaction). OR_adj_ 95% CI: Compared to reference category, adjusted odds ratio (OR_adj_) and 95% confidence intervals (95% CI) adjusted for sex, age, education level, seniority, occupation, establishment size, activity sector and economic activity. * indicates exposure OR_adj_ statistically different from 1 (α < 0.05) for row year; BY (Between Years difference): compares between row year and previous row year (i.e., 2010 vs. 2005 or 2015 vs. 2010); ↓ indicates no overlapping OR_adj_ 95% CI and decrease of OR_adj_, and ↑ indicates no overlapping OR_adj_ 95% CI and increase of OR_adj._

**Table 2:** Relationship between well-being and supervising others, long working hours and other paid job in 2005–2015.

DepVariable	Year	Supervising Others 1 or More (Reference: None)		Long Working Hours Yes (Reference: No)		Other Paid Job(s) Yes (Reference: No Other Paid Job )	
		OR_adj_ 95% CI	BY	OR_adj_ 95% CI	BY	OR_adj_ 95% CI	BY
Anxiety	2005	0.87 (0.76;1.00)		2.08 (1.81;2.39) *		1.20 (0.97;1.49)	
	2010	1.16 (1.01;1.34) *	↑	2.06 (1.78;2.39) *		1.07 (0.85;1.34)	
	2015	1.38 (1.24;1.52) *		2.41 (2.16;2.68) *		1.19 (1.02;1.38) *	
Fatigue	2005	0.89 (0.81;0.98) *		2.39 (2.17;2.63) *		1.43 (1.25;1.65) *	
	2010	1.04 (0.95;1.14)		2.40 (2.18;2.63) *		1.12 (0.98;1.29)	
	2015	1.24 (1.14;1.34) *	↑	2.16 (1.98;2.36) *		1.04 (0.92;1.18)	
Dissatisfacion	2005	0.65 (0.58;0.73) *		1.46 (1.30;1.63) *		1.45 (1.24;1.68) *	
	2010	0.59 (0.52;0.66) *		1.50 (1.34;1.67) *		1.19 (1.03;1.38) *	
	2015	0.76 (0.69;0.84) *	↑	1.57 (1.42;1.74) *		1.22 (1.07;1.39) *	

N weighted supervising: 67685 (anxiety); 67695 (fatigue); 68884 (dissatisfaction). Long working: 67158 (anxiety); 67170 (fatigue); 68256 (dissatisfaction). Other paid: 67976 (anxiety); 67986 (fatigue); 69146 (dissatisfaction). OR_adj_ 95% CI: Compared to reference category, adjusted odds ratio (OR_adj_) and 95% confidence intervals (95% CI) adjusted for sex, age, education level, seniority, occupation, establishment size, activity sector and economic activity. * indicates exposure OR_adj_ statistically different from 1 (α < 0.05) for row year; BY (Between Years difference): compares between row year and previous row year (i.e., 2010 vs. 2005 or 2015 vs. 2010); ↓ indicates no overlapping OR_adj_ 95% CI and decrease of OR_adj_, and ↑ indicates no overlapping OR_adj_ 95% CI and increase of OR_adj._

**Table 3:** Relationship between well-being and the number of weekly working hours in 2005–2015. Hours usually work per week in main paid job (reference: 36 h–45 h).

DepVariable	Year	>=45 h		20 h–35 h		<20 h	
		ORadj 95% CI	BY	ORadj 95% CI	BY	ORadj 95% CI	BY
Anxiety	2005	1.69 (1.48;1.94) *		1.07 (0.93;1.23)		0.55 (0.41;0.75) *	
	2010	1.63 (1.40;1.89) *		1.09 (0.95;1.25)		0.79 (0.61;1.03)	
	2015	2.26 (2.03;2.53) *	↑	1.46 (1.32;1.62) *	↑	1.10 (0.93;1.30)	
Fatigue	2005	2.10 (1.92;2.30) *		0.84 (0.76;0.93) *		0.48 (0.40;0.58) *	
	2010	2.01 (1.84;2.20) *		1.07 (0.98;1.17)	↑	0.84 (0.71;0.98) *	↑
	2015	1.90 (1.74;2.07) *		1.15 (1.06;1.24) *		0.82 (0.71;0.94) *	
Dissatisfacion	2005	1.44 (1.30;1.60) *		0.88 (0.79;0.97) *		0.69 (0.57;0.82) *	
	2010	1.50 (1.36;1.66) *		1.04 (0.95;1.14)		0.75 (0.63;0.88) *	
	2015	1.55 (1.41;1.71) *		1.15 (1.05;1.25) *		0.90 (0.78;1.04)	

N weighted: 67131 (anxiety); 67143 (fatigue); 68252 (dissatisfaction).OR_adj_ 95% CI: Compared to reference category, adjusted odds ratio (OR_adj_) and 95% confidence intervals (95% CI) adjusted for sex, age, education level, seniority, occupation, establishment size, activity sector and economic activity. * indicates exposure OR_adj_ statistically different from 1 (α < 0.05) for row year; BY (Between Years difference): compares between row year and previous row year (i.e., 2010 vs. 2005 or 2015 vs. 2010); ↓ indicates no overlapping OR_adj_ 95% CI and decrease of OR_adj_, and ↑ indicates no overlapping OR_adj_ 95% CI and increase of OR_adj._

**Table 4:** Relationship between well-being and atypical working days, shift work and alternating/rotating shifts in 2005–2015.

DepVariable	Year	Work on Saturdays and/or Sundays Yes (Reference: No)		Work in Shits Yes (Reference: No)		Alternating or Rotating Shift Yes (Reference: No)	
		ORadj 95% CI	BY	ORadj 95% CI	BY	ORadj 95% CI	BY
Anxiety	2005	1.64 (1.46;1.83) *		1.37 (1.20;1.56) *		1.16 (0.97;1.39)	
	2010	1.53 (1.36;1.72) *		1.74 (1.52;1.99) *		1.23 (1.02;1.48) *	
	2015	1.60 (1.47;1.74) *		1.17 (1.06;1.29) *	↓	1.18 (1.04;1.34) *	
Fatigue	2005	1.81 (1.68;1.95) *		1.46 (1.34;1.59) *		1.27 (1.13;1.43) *	
	2010	1.58 (1.47;1.70) *		1.46 (1.34;1.59) *		1.47 (1.31;1.65) *	
	2015	1.91 (1.78;2.04) *	↑	1.44 (1.34;1.56) *		1.36 (1.24;1.50) *	
Dissatisfacion	2005	1.40 (1.29;1.52) *		1.57 (1.43;1.72) *		1.60 (1.42;1.80) *	
	2010	1.42 (1.31;1.53) *		1.48 (1.36;1.62) *		1.47 (1.31;1.65) *	
	2015	1.56 (1.45;1.68) *		1.47 (1.36;1.59) *		1.44 (1.30;1.60) *	

N weighted work on Saturdays/sundays: 67218 (anxiety); 67228 (fatigue); 68343 (dissatisfaction); work in shifts: 67913 (anxiety); 67923 (fatigue); 69064 (dissatisfaction); alternating/rotating shifts: 67788 (anxiety); 67798 (fatigue); 68941 (dissatisfaction). OR_adj_ 95% CI: Compared to reference category, adjusted odds ratio (OR_adj_) and 95% confidence intervals (95% CI) adjusted for sex, age, education level, seniority, occupation, establishment size, activity sector and economic activity. * indicates exposure OR_adj_ statistically different from 1 (α < 0.05) for row year; BY (Between Years difference): compares between row year and previous row year (i.e., 2010 vs. 2005 or 2015 vs. 2010); ↓ indicates no overlapping OR_adj_ 95% CI and decrease of OR_adj_, and ↑ indicates no overlapping OR_adj_ 95% CI and increase of OR_adj._

## Appendix A

SPSS syntax details for variables’ transformation.

* data filter: years 2005, 2010 or 2015 and employees or self-employees in EUR-27

FILTER OFF.

USE ALL.

SELECT IF (year >= 2005 & EU27=1).

EXECUTE.

USE ALL.

COMPUTE filtro1=(y15_Q2c=1 OR y10_hh2d=1).

VARIABLE LABELS filtro1 ’((y15_Q2c=1 OR y10_hh2d=1))(FILTER)’.

VALUE LABELS filtro1 0 ’Not Selected’ 1 ’Selected’.

FORMATS filtro1 (f1.0).

EXECUTE.

USE ALL.

SELECT IF (filtro1=1).

EXECUTE.

*moderator variables for GLM.

RECODE year (2005=0) (2010=1) (2015=0) (ELSE=SYSMIS) INTO y10_05.

VARIABLE LABELS y10_05 ’comparar 2005 con 2010 Year 2005 o 15 is 0, 2010 is 1 else sysmis’.

EXECUTE.

RECODE year (2005=0) (2010=0) (2015=1) (ELSE=SYSMIS) INTO y15_05.

VARIABLE LABELS y15_05 ’comparar 2005 con 2015 Year 2015 es 1 y 05 o 2010 is 0 else sysmis’.

EXECUTE.

RECODE year (2005=1) (2010=0) (2015=0) (ELSE=SYSMIS) INTO y05_10.

VARIABLE LABELS y05_10 ’comparar 2010 con 2005 Year 2005 es1 y 15 2010 is 0 else sysmis’.

EXECUTE.

RECODE year (2005=0) (2010=0) (2015=1) (ELSE=SYSMIS) INTO y15_10.

VARIABLE LABELS y15_10 ’comparar 2010 con 2015 (2005=0) (2010=0) (2015=1) else sysmis’.

EXECUTE.

RECODE year (2005=1) (2010=0) (2015=0) (ELSE=SYSMIS) INTO y05_15.

VARIABLE LABELS y05_15 ’comparar 2015 con 2005 (2005=1) (2010=0) (2015=0)else sysmis’.

EXECUTE.

RECODE year (2005=0) (2010=1) (2015=0) (ELSE=SYSMIS) INTO y10_15.

VARIABLE LABELS y10_15 ’comparar 2005 con 2010 (2005=0) (2010=1) (2015=0) else sysmis’.

EXECUTE.

* Oucome variables.

DO IF (year=2005).

RECODE y15_Q78h_lt y15_Q78i_lt (SYSMIS=0) (2=0) (ELSE=Copy) INTO anxiety05 fatigue05.

VARIABLE LABELS anxiety05 ’anxiety clonada como en 2005’ /fatigue05 ’fatigue clonada como en 2005’.

END IF.

EXECUTE.

DO IF (year>2005 & y15_Q74_lt=1).

RECODE y15_Q78h_lt y15_Q78i_lt (2=0) (8=0)(9=0)(ELSE=Copy) INTO anxiety05 fatigue05.

VARIABLE LABELS anxiety05 ’anxiety clonada como en 2005’ /fatigue05 ’fatigue clonada como en 2005’.

END IF.

DO IF (year>2005 & y15_Q74_lt=2).

RECODE y15_Q78h_lt y15_Q78i_lt (SYSMIS=0) (ELSE=0) INTO anxiety05 fatigue05.

VARIABLE LABELS anxiety05 ’anxiety clonada como en 2005’ /fatigue05 ’fatigue clonada como en 2005’.

END IF.

VALUE LABELS anxiety05 0 ’Not mentioned’ 1 ’Mentioned’.

VALUE LABELS fatigue05 0 ’Not mentioned’ 1 ’Mentioned’.

EXECUTE.

RECODE y15_Q88 (MISSING=SYSMIS) (3 thru Highest=1) (Lowest thru 2=0) INTO insatisfactionDic.

VARIABLE LABELS insatisfactionDic ’Insatisfaction dicotomica’.

VALUE LABELS insatisfactionDic 0 ’Satisfied’ 1 ’Not satisfied’.

EXECUTE.

*exposure variables.

COMPUTE contract=$SYSMIS.

VARIABLE LABELS contract ’fusion Y15_Q7_lt y15_Q11_lt autonomos y diferentes categorias de autoempleo y duracion de contrato’.

VALUE LABELS contract 1 ’Indefinite contract’ 2 ’Temporary’ 3 ’Self-Employed’ 4 ’No contract & other’.

EXECUTE.

IF ANY(Y15_Q7_lt, 1,2) contract = 4.

IF (y15_Q11_lt=1) contract = 1.

IF ANY(y15_Q11_lt, 2,3,4) contract = 2.

IF (y15_Q11_lt=5) contract = 4.

IF (Y15_Q7_lt=2) contract = 3.

*estas dos no se ejecutna en realidad porque son missings.

IF (y15_Q11_lt=8) contract= 8.

IF (y15_Q11_lt=9) contract= 9.

execute.

COMPUTE supervision=$SYSMIS.

VARIABLE LABELS supervision ’personas a tu cargo recodificacion 0-1 ’.

VALUE LABELS supervision 0 ’none’ 1 ’1 or more’.

EXECUTE.

IF ANY(y15_Q23_lt,2,3) supervision = 1.

IF (y15_Q23_lt=1) supervision= 0.

COMPUTE hoursworked=$SYSMIS.

VARIABLE LABELS hoursworked ’Horas duracion jornada ’.

VALUE LABELS hoursworked 1 ’36h…45h’ 2 ’20h...35h’ 3 ’<20h’ 4 ’>=45h’.

EXECUTE.

IF (y15_Q24<20) hoursworked = 3.

IF (y15_Q24>=20 AND y15_Q24<=35) hoursworked = 2.

IF (y15_Q24>35 AND y15_Q24<=45) hoursworked = 1.

IF (y15_Q24>45) hoursworked = 4.

COMPUTE longworkhour=$SYSMIS.

VARIABLE LABELS longworkhour ’Long working hours si y15_Q37d es un dia o mas y hoursworked mas de 45 horas semana ’.

VALUE LABELS longworkhour 0 ’No’ 1 ’Yes’.

EXECUTE.

IF (y15_Q37d<1 OR y15_Q24<=45) longworkhour = 0.

IF (y15_Q37d>=1 AND y15_Q24<=45) longworkhour = 0.

IF (y15_Q37d>=1 AND y15_Q24>45) longworkhour = 1.

COMPUTE workweekend=$SYSMIS.

VARIABLE LABELS workweekend ’Work on Saturdays and/or Sundays ’.

VALUE LABELS workweekend 0 ’No’ 1 ’Yes’.

EXECUTE.

IF (y15_Q37b<1 AND y15_Q37c<1) workweekend = 0.

IF (y15_Q37b>=1 OR y15_Q37c>=1) workweekend = 1.

RECODE y15_Q39e (MISSING=SYSMIS) (1=1) (2=0) INTO shifts.

VARIABLE LABELS shifts ’Work in shits (reference: No) recodificada original’.

VALUE LABELS shifts 0 ’No’ 1 ’Yes’.

EXECUTE.

COMPUTE alternateshift=$SYSMIS.

VARIABLE LABELS alternateshift ’Alternating or rotating shift (reference: No) ’.

VALUE LABELS alternateshift 0 ’No’ 1 ’Yes’.

EXECUTE.

IF (y15_Q39e=2 OR y15_Q41<=2) alternateshift = 0.

IF (y15_Q41 =3 )    alternateshift = 1.

IF (y15_Q41 =4 )    alternateshift = 0.

RECODE y15_Q27_lt (MISSING=SYSMIS) (4=SYSMIS) (1=1) (2=0) INTO otherpaid.

VARIABLE LABELS otherpaid ’Other paid job(s) (reference: No other paid job)’.

VALUE LABELS otherpaid 0 ’No’ 1 ’Yes’.

EXECUTE.

* Additional variables.

RECODE y15_Q2a (MISSING=SYSMIS) (1=1) (2=2) INTO sex.

VARIABLE LABELS sex ’sex (male/female)’.

VALUE LABELS sex 1 ’men’ 2 ’women’.

EXECUTE.

RECODE y15_Q2b (MISSING=SYSMIS) (Lowest thru 24=1) (25 thru 34=2) (35 thru 54=3) (55 thru

 Highest=4) INTO age.

VARIABLE LABELS age ’age (16-24; 25-34; 35-54; 55 or more)’.

VALUE LABELS age 1 ’16-24’ 2 ’25-34’ 3 ’35-54’ 4 ’55 or more’.

EXECUTE.

RECODE y15_ISCED_lt (SYSMIS=SYSMIS) (Lowest thru 1=1) (2 thru 4=2) (5 thru 6=3) (ELSE=SYSMIS) INTO

 educa.

VARIABLE LABELS educa ’education level’.

VALUE LABELS educa 1 ’pre-primary or primary’ 2 ’lower, upper and post-secondary’ 3 ’tertiary education, first and second’.

EXECUTE.

RECODE y15_Q17_lt (MISSING=SYSMIS) (Lowest thru 1=1) (1 thru 5=2) (5 thru Highest=3) INTO seniority.

VARIABLE LABELS seniority ’seniority’.

VALUE LABELS seniority 1 ’1 year or less’ 2 ’more than 1 to 5 years’ 3 ’more than 5 years’.

EXECUTE.

DO IF (year=2015).

RECODE y15_Q16b (MISSING=SYSMIS) (1 thru 2=1) (3=2) (4=3) (7 thru Highest=SYSMIS) INTO estabsize.

END IF.

EXECUTE.

DO IF (year<=2010).

RECODE y15_Q16a (MISSING=SYSMIS) (1 thru 3=1) (4 thru 6=2) (7 thru 8=3) (9 thru Highest=SYSMIS) INTO estabsize.

END IF.

VARIABLE LABELS estabsize ’establishment size (number of workers in the workplace)’.

VALUE LABELS estabsize 1 ’1-9 ’ 2 ’10-249’ 3 ’250 and over’.

EXECUTE.

RECODE y15_Q14 (MISSING=SYSMIS) (1=1) (2=2) (3 thru 5=3) INTO sector.

VARIABLE LABELS sector ’activity sector (public; private; other)’.

VALUE LABELS sector 1 ’private’ 2 ’public’ 3 ’other’.

EXECUTE.

RECODE y15_nace_r1_lt_4 (MISSING=SYSMIS) (ELSE=Copy) INTO activity.

VARIABLE LABELS activity ’economic activity’.

VALUE LABELS activity 1 ’agriculture’ 2 ’industry’ 3 ’services’ 4 ’public administration and defence’.

EXECUTE.

RECODE y15_ISCO_88_1 (MISSING=SYSMIS) (ELSE=Copy) INTO occupation.

VARIABLE LABELS occupation ’occupation as main paid job’.

EXECUTE.

missing values contract to occupation (8,9).

## Appendix B

SPSS syntax details for logistic regressions.

*Macro.

DEFINE !MacrGLMdic (response= !TOKENS(1) / predictor= !TOKENS(1) / ajuste= !TOKENS(8) )

GENLIN !response (REFERENCE=FIRST) BY y10_05 y15_05 !predictor !ajuste (ORDER=DESCENDING)

 /MODEL y10_05 !predictor y15_05 y10_05*!predictor y15_05*!predictor !ajuste INTERCEPT=YES

 /DISTRIBUTION=BINOMIAL LINK=LOGIT

 /CRITERIA METHOD=FISHER(1) SCALE=1 COVB=MODEL MAXITERATIONS=200 MAXSTEPHALVING=15

 PCONVERGE=1E-006(ABSOLUTE) SINGULAR=1E-012 ANALYSISTYPE=3(WALD) CILEVEL=95 CITYPE=WALD

 LIKELIHOOD=FULL

 /MISSING CLASSMISSING=EXCLUDE

 /PRINT CPS DESCRIPTIVES MODELINFO FIT SUMMARY SOLUTION (EXPONENTIATED).

GENLIN !response (REFERENCE=FIRST) BY y05_10 y15_10 !predictor !ajuste (ORDER=DESCENDING)

 /MODEL y05_10 !predictor y15_10 y05_10*!predictor y15_10*!predictor !ajuste INTERCEPT=YES

 DISTRIBUTION=BINOMIAL LINK=LOGIT

 /CRITERIA METHOD=FISHER(1) SCALE=1 COVB=MODEL MAXITERATIONS=200 MAXSTEPHALVING=15

 PCONVERGE=1E-006(ABSOLUTE) SINGULAR=1E-012 ANALYSISTYPE=3(WALD) CILEVEL=95 CITYPE=WALD

 LIKELIHOOD=FULL

 /MISSING CLASSMISSING=EXCLUDE

 /PRINT CPS DESCRIPTIVES MODELINFO FIT SUMMARY SOLUTION (EXPONENTIATED).

GENLIN !response (REFERENCE=FIRST) BY y05_15 y10_15 !predictor !ajuste (ORDER=DESCENDING)

 /MODEL y05_15 !predictor y10_15 y05_15*!predictor y10_15*!predictor !ajuste INTERCEPT=YES

 DISTRIBUTION=BINOMIAL LINK=LOGIT

 /CRITERIA METHOD=FISHER(1) SCALE=1 COVB=MODEL MAXITERATIONS=200 MAXSTEPHALVING=15

 PCONVERGE=1E-006(ABSOLUTE) SINGULAR=1E-012 ANALYSISTYPE=3(WALD) CILEVEL=95 CITYPE=WALD

 LIKELIHOOD=FULL

 /MISSING CLASSMISSING=EXCLUDE

 /PRINT CPS DESCRIPTIVES MODELINFO FIT SUMMARY SOLUTION (EXPONENTIATED).

!ENDDEFINE.

*analysis.

WEIGHT BY w5_eu27.

USE ALL.

 !MacrGLMdic response=anxiety05 predictor=contract ajuste= sex age educa seniority estabsize sector activity occupation.

 !MacrGLMdic response=fatigue05 predictor=contract ajuste= sex age educa seniority estabsize sector activity occupation.

 !MacrGLMdic response=insatisfactionDic predictor=contract ajuste= sex age educa seniority estabsize sector activity occupation.

!MacrGLMdic response=anxiety05 predictor=supervision ajuste= sex age educa seniority estabsize sector activity occupation.

 !MacrGLMdic response=fatigue05 predictor=supervision ajuste= sex age educa seniority estabsize sector activity occupation.

 !MacrGLMdic response=insatisfactionDic predictor=supervision ajuste= sex age educa seniority estabsize sector activity occupation.

!MacrGLMdic response=anxiety05 predictor=hoursworked ajuste= sex age educa seniority estabsize sector activity occupation.

 !MacrGLMdic response=fatigue05 predictor=hoursworked ajuste= sex age educa seniority estabsize sector activity occupation.

 !MacrGLMdic response=insatisfactionDic predictor=hoursworked ajuste= sex age educa seniority estabsize sector activity occupation.

!MacrGLMdic response=anxiety05 predictor=longworkhour ajuste= sex age educa seniority estabsize sector activity occupation.

 !MacrGLMdic response=fatigue05 predictor=longworkhour ajuste= sex age educa seniority estabsize sector activity occupation.

 !MacrGLMdic response=insatisfactionDic predictor=longworkhour ajuste= sex age educa seniority estabsize sector activity occupation.

!MacrGLMdic response=anxiety05 predictor=workweekend ajuste= sex age educa seniority estabsize sector activity occupation.

 !MacrGLMdic response=fatigue05 predictor=workweekend ajuste= sex age educa seniority estabsize sector activity occupation.

 !MacrGLMdic response=insatisfactionDic predictor=workweekend ajuste= sex age educa seniority estabsize sector activity occupation.

!MacrGLMdic response=anxiety05 predictor=shifts ajuste= sex age educa seniority estabsize sector activity occupation.

 !MacrGLMdic response=fatigue05 predictor=shifts ajuste= sex age educa seniority estabsize sector activity occupation.

 !MacrGLMdic response=insatisfactionDic predictor=shifts ajuste= sex age educa seniority estabsize sector activity occupation.

!MacrGLMdic response=anxiety05 predictor=alternateshift ajuste= sex age educa seniority estabsize sector activity occupation.

 !MacrGLMdic response=fatigue05 predictor=alternateshift ajuste= sex age educa seniority estabsize sector activity occupation.

 !MacrGLMdic response=insatisfactionDic predictor=alternateshift ajuste= sex age educa seniority estabsize sector activity occupation.

!MacrGLMdic response=anxiety05 predictor=otherpaid ajuste= sex age educa seniority estabsize sector activity occupation.

 !MacrGLMdic response=fatigue05 predictor=otherpaid ajuste= sex age educa seniority estabsize sector activity occupation.

 !MacrGLMdic response=insatisfactionDic predictor=otherpaid ajuste= sex age educa seniority estabsize sector activity occupation.

## Appendix C

**Table A1:** Definition of all variables and descriptive statistics for each year.

Variable	Response Categories	2005	2010	2015
Anxiety: “Over the last 12 months, did you have anxiety?”	No	22582	30335	28678
	Yes	1944	1729	3024
Fatigue: “Over the last 12 months, did you have overall fatigue?”	No	19019	26388	25823
	Yes	5506	5687	5879
Job satisfaction: “On the whole, are you very satisfied, satisfied, not very satisfied, or not at all satisfied with the working conditions in your main paid job?”	Very satisfied	6143	8268	8517
	Satisfied	13970	19618	19568
	Not very satisfied	3395	4239	3724
	Not at all satisfied	835	827	792
Type of employment contract and self-employment. Permanent contract (unlimited duration); Temporary contract (limited duration, fixed-term, temporary employment agency contract) Self-employed (Having more than one client, not receiving regular (monthly) payment, resources should come through payment for products or services provided and being able to take decisions for organising work and recruiting staff); No contract & others (apprenticeship or other training scheme, no contract and others)	Permanent contract	15630	22458	22702
	Temporary contract	2922	3663	3766
	Self-employed	4030	5096	4913
	No contract & others	1656	1567	1341
Supervision: “How many people work under your supervision, for whom pay increases, bonuses or promotion depend directly on you? “	No subordinates	19651	27098	26885
	One or more	4380	5747	5459
Weekly working hours: “How many hours do you usually work per week in your main paid job? “	Less than 20 h	1594	2202	2734
	Between 20 h and 35 h	5264	7799	7524
	between 36 h and 45 h	12627	16975	16474
	More than 45 h	4433	5441	5122
Long working hours: working more than 45 h per week and more than 10 h per day at least once a month	No	20569	28527	28171
	Yes	3251	3922	3800
Atypical working days: working on Saturday or Sunday one or more times a month	No	10911	15506	14854
	Yes	13020	17184	17208
Shift work: any kind of shift arrangement	No	19965	27210	25709
	Yes	4245	5753	6907
Alternating/rotating shifts: shift arrangement different of daily split shifts (with a break of at least 4 h in between) or permanent shifts (morning, afternoon or night)	No	22060	30051	29217
	Yes	2122	2871	3310
Other Paid Jobs: “besides Your Main Paid Job, do You have any other Paid Job(s)?”	No	22772	30657	30171
	Yes	1480	2285	2449
Sex	Men	13680	18280	17155
	Women	10846	14909	15576
Age	16–24	2676	2615	2151
	25–34	5614	7644	6702
	35–54	13104	18120	17809
	55 or more	3048	4653	5926
Education level	Pre-primary or primary	1483	1375	883
	Lower, upper and post-secondary	16670	17991	21155
	Tertiary education, first and second	6270	8014	10545
Seniority	1 year or less	4276	4993	5397
	More than 1 to 5 years	7149	9870	8525
	More than 5 years	12597	17930	18205
Establishment size (number of workers in the workplace)	1–9	8679	13139	10002
	10–249	11101	15018	11201
	250 and over	3551	4019	10,063
Activity sector	Private	16551	23381	23315
	Public	6002	7470	7056
	Other	1692	2153	2196
Economic activity	Agriculture	1184	1447	1209
	Industry	7102	8250	7530
	Services	8675	12349	13285
	Public Administration	7250	10746	10450
Occupation	Managers	2206	3002	2467
	Professionals	3622	4938	5611
	Technicians and associate professionals	3223	5588	4842
	Clerical support workers	2908	3618	3727
	Service and sales workers	3117	4569	4982
	Craft and related trades workers	3465	4361	3982
	Plant and machine operators, and assemblers	2032	2840	2693
	Elementary occupations	2796	3036	3212
Total (differences between Total and sum of categories are missing values)		24526	33190	32738

## References

[1] Eurofound and EU-OSHA (2014). Psychosocial Risks in Europe. Prevalence and Strategies for Prevention.

[2] LaMontagne A.D., Milner A., Krnjacki L., Schlichthorst M., Kavanagh A., Page K., Pirkis J. (2016). Psychosocial job quality, mental health, and subjective wellbeing: A cross-sectional analysis of the baseline wave of the Australian Longitudinal Study on Male Health. BMC Public Health.

[3] Stansfeld S., Candy B. (2006). Psychosocial work environment and mental health: A meta-analytic review. Scand. J. Work Environ. Health.

[4] Van der Doef M., Maes S. (1999). The job demand-control (-support) model and psychological well-being: A review of 20 years of empirical research. Work Stress.

[5] Nappo N. (2019). Is there an association between working conditions and health? An analysis of the Sixth European Working Conditions Survey data. PLoS ONE.

[6] Nappo N. (2018). Working conditions and anxiety. An analysis on the sixth european working conditions survey data. Int. J. Humanit. Soc. Sci..

[7] Malard L., Chastang J.F., Schütte S., Parent-Thirion A., Vermeylen G., Niedhammer I. (2013). Changes in psychosocial work exposures among employees between 2005 and 2010 in 30 countries in Europe. J. Occup. Environ. Med..

[8] Benach J., Gimeno D., Benavides F.G., Martínez J.M., Torné M. (2004). Types of employment and health in the European Union: Changes from 1995 to 2000. Eur. J. Public Health.

[9] Erhel C., Guergoat-Larivière M., Leschke J., Watt A. (2012). Trends in Job Quality during the Great Recession: A Comparative Approach for the EU. Working Paper nº 161-1. https://halshs.archives-ouvertes.fr/halshs-00966898.

[10] Wood S., Ogbonnaya C. (2018). High-involvement management, economic recession, well-being, and organizational performance. J. Manag..

[11] Rostila M. (2008). The Swedish labour market in the 1990s: The very last of the healthy jobs?. Scand. J. Public Health.

[12] Ardito C., D’Errico A., Leombruni R., Pacelli L. (2012). Health and Well-Being at Work: A Report Based on the Fifth European Working Conditions Survey.

[13] Julià M., Vanroelen C., Bosmans K., van Aerden K., Benach J. (2017). Precarious employment and quality of employment in relation to health and well-being in Europe. Int. J. Health Serv..

[14] Parent-Thirion A., Vermeylen G., Houten G., Lyly-Yrjänäinen M., Biletta I., Cabrita J. (2012). Fifth European Working Conditions Survey.

[15] Robert G., Martínez J.M., García A.M., Benavides F.G., Ronda E. (2014). From the boom to the crisis: Changes in employment conditions of immigrants in Spain and their effects on mental health. Eur. J. Public Health.

[16] Utzet M., Moncada S., Molinero E., Llorens C., Moreno N., Navarro A. (2014). The changing patterns of psychosocial exposures at work in the South of Europe: Spain as a labor market laboratory. Am. J. Ind. Med..

[17] Utzet M., Navarro A., Llorens C., Muntaner C., Moncada S. (2016). Is the worsening of psychosocial exposures associated with mental health? Comparing two population-based cross-sectional studies in Spain, 2005–2010. Am. J. Ind. Med..

[18] Blank C., Gatterer K., Leichtfried V., Pollhammer D., Mair-Raggautz M., Duschek S., Humpeler E., Schobersberger W. (2018). Short vacation improves stress-level and well-being in german-speaking middle-managersa randomized controlled trial. Int. J. Environ. Res. Public Health.

[19] Maharaj S., Lees T., Lal S. (2019). Prevalence and risk factors of depression, anxiety, and stress in a cohort of australian nurses. Int. J. Environ. Res. Public Health.

[20] Wong V., Au-Yeung T.C. (2019). Expediting youth’s entry into employment whilst overlooking precariousness: Flexi-employability and disciplinary activation in Hong Kong. Soc. Policy Adm..

[21] Ronda E., Briones-Vozmediano E., Galon T., Garcia A.M., Benavides F.G., Agudelo-Suarez A.A. (2016). A qualitative exploration of the impact of the economic recession in spain on working, living and health conditions: Reflections based on immigrant workers’ experiences. Health Expect..

[22] Sorensen G., Peters S., Nielsen K., Nagler E., Karapanos M., Wallace L., Burke L., Dennerlein J.T., Wagner G.R. (2019). Improving working conditions to promote worker safety, health, and wellbeing for low-wage workers: The workplace organizational health study. Int. J. Environ. Res. Public Health.

[23] Berglund T. (2014). Crisis and quality of work in the Nordic employment regime. Int. Rev. Sociol..

[24] Chambel M.J., Farina A. (2015). HRM and temporary workers’ well-being: A study in Portugal and Brazil. Int. J. Cross Cult. Manag..

[25] Benach J., Vanroelen C., Vives A., de Witte H. (2013). Quality of Employment Conditions and Employment Relations in Europe.

[26] Schutte S., Chastang J.F., Malard L., Parent-Thirion A., Vermeylen G., Niedhammer I. (2014). Psychosocial working conditions and psychological well-being among employees in 34 European countries. Arch. Occup. Environ. Health.

[27] Lorente L., Tordera N., Peiro J.M. (2018). How work characteristics are related to european workers’ psychological well-being. A comparison of two age groups. Int. J. Environ. Res. Public Health.

[28] Meyer S.C., Hunefeld L. (2018). Challenging cognitive demands at work, related working conditions, and employee well-being. Int. J. Environ. Res. Public Health.

[29] Van Aerden K., Puig-Barrachina V., Bosmans K., Vanroelen C. (2016). How does employment quality relate to health and job satisfaction in europe? A typological approach. Soc. Sci. Med..

[30] Pieper C., Schroer S., Eilerts A.L. (2019). Evidence of workplace interventions-a systematic review of systematic reviews. Int. J. Environ. Res. Public Health.

[31] Welz C., Vargas O., Broughton A., Van Gyes G., Szekér L., Curtarelli M., Fric K., Kerckhofs P., Diemu-Trémolières S. (2014). Impact of the Crisis on Industrial Relations and Working Conditions in Europe.

[32] Lopes H., Lagoa S., Calapez T. (2014). Work autonomy, work pressure, and job satisfaction: An analysis of European Union countries. Econ. Labour Relat. Rev..

[33] Curtarelli M., Fric K., Vargas O., Welz C. (2014). Job quality, industrial relations and the crisis in Europe. Int. Rev. Sociol..

[34] Piasna A. (2018). Scheduled to work hard: The relationship between non-standard working hours and work intensity among european workers (2005–2015). Hum. Resour. Manag. J..

[35] Boada-Grau J., Robert-Sentís L., Gil-Ripoll C., Vigil-Colet A. (2013). Desarrollo, consistencia interna, fiabilidad y validez de una escala de riesgos laborales en lengua Española [Development, internal consistency, reliability and validity of a scale of occupational hazards in Spanish.]. An. Psicol..

[36] Morley J. (2014). Policy Lessons from the Fifth EWCS: The Pursuit of More and Better Jobs.

[37] Bergström O. (2018). Changing restructuring regimes in 11 European countries during and after the financial crisis. Eur. J. Ind. Relat..

[38] Guest D.E., Isaksson K. (2019). Temporary employment contracts and employee well-being during and after the financial crisis: Introduction to the special issue. Econ. Ind. Democr..

[39] Parent-Thirion A., Biletta I., Cabrita J., Vargas Llave O., Vermeylen G., Wilczyńska A., Wilkens M. (2019). Sixth European Working Conditions Survey—Overview Report.

[40] Eurofound (2010). Changes over Time. First Findings from the Fifth European Working Conditions Survey.

[41] Benavides F.G., Benach J., Diez-Roux A.V., Roman C. (2000). How do types of employment relate to health indicators? Findings from the second European survey on working conditions. J. Epidemiol. Community Health.

[42] Eurofound (2013). Impact of the Crisis on Working Conditions in Europe.

[43] Artazcoz L., Cortès I., Puig-Barrachina V., Benavides F.G., Escribà-Agüir V., Borrell C. (2014). Combining employment and family in Europe: The role of family policies in health. Eur. J. Public Health.

[44] Bartoll X., Cortès I., Artazcoz L. (2014). Full- and part-time work: Gender and welfare-type differences in european working conditions, job satisfaction, health status, and psychosocial issues. Scand. J. Work Environ. Health.

[45] Muckenhuber J., Burkert N., Großschädl F., Freidl W. (2014). Income inequality as a moderator of the relationship between psychological job demands and sickness absence, in particular in men: An international comparison of 23 countries. PLoS ONE.

[46] Eurofound (2007). Quality Report of the Fourth European Working Conditions Survey.

[47] Eurofound (2010). Quality Assurance Report. Fifth European Working Conditions Survey.

[48] Parent-Thirion A., Fernández E., Hurley J., Vermeylen G. (2007). Fourth European Working Conditions Survey.

[49] Eurostat (2016). Statistical Office of the European Communities: EU Labour Force Survey. http://ec.europa.eu/eurostat/statisticsexplained/index.php/EU_labour_force_survey_-_methodology.

[50] European Foundation for the Improvement of Living and Working Conditions (2018). European Working Conditions Survey Integrated Data File, 1991–2015.

[51] Houdmont J., Jachens L., Randall R., Hopson S., Nuttall S., Pamia S. (2019). What does a single-item measure of job stressfulness assess?. Int. J. Environ. Res. Public Health.

[52] Lepold A., Tanzer N., Bregenzer A., Jimenez P. (2018). The efficient measurement of job satisfaction: Facet-items versus facet scales. Int. J. Environ. Res. Public Health.

[53] Schwendimann R., Dhaini S., Ausserhofer D., Engberg S., Zuniga F. (2016). Factors associated with high job satisfaction among care workers in swiss nursing homes—A cross sectional survey study. BMC Nurs..

[54] Dowler K. (2016). Job satisfaction, burnout, and perception of unfair treatment: The relationship between race and police work. Police Q..

[55] Kinzl J.F., Knotzer H., Traweger C., Lederer W., Heidegger T., Benzer A. (2005). Influence of working conditions on job satisfaction in anaesthetists. Br. J. Anaesth..

[56] EMCONET (2007). Employment Conditions and Health Inequalities. http://cdrwww.who.int/entity/social_determinants/resources/articles/emconet_who_report.pdf.

[57] Van Aerden K., Moors G., Levecque K., Vanroelen C. (2014). Measuring employment arrangements in the European labour force: A typological approach. Soc. Indic. Res..

[58] Brydsten A., Hammarström A., San Sebastian M. (2016). The impact of economic recession on the association between youth unemployment and functional somatic symptoms in adulthood: A difference-in-difference analysis from Sweden. BMC Public Health.

[59] Wagenaar A.F., Kompier M.A.J., Houtman I.L.D., van den Bossche S.N.J., Taris T.W. (2012). Impact of employment contract changes on workers’ quality of working life, job insecurity, health and work-related attitudes. J. Occup. Health.

[60] De Moortel D., Thevenon O., De Witte H., Vanroelen C. (2017). Working hours mismatch, macroeconomic changes, and mental well-being in Europe. J. Health Soc. Behav..

[61] Angrave D., Charlwood A. (2015). What is the relationship between long working hours, over-employment, under-employment and the subjective well-being of workers? Longitudinal evidence from the UK. Hum. Relat..

[62] Arlinghaus A., Bohle P., Iskra-Golec I., Jansen N., Jay S., Rotenberg L. (2019). Working time society consensus statements: Evidence-based effects of shift work and non-standard working hours on workers, family and community. Ind. Health.

[63] Julia M., Belvis F., Vives A., Tarafa G., Benach J. (2019). Informal employees in the European Union: Working conditions, employment precariousness and health. J. Public Health.

[64] Lofstrom M. (2013). Does self-employment increase the economic well-being of low-skilled workers?. Small Bus. Econ..

[65] Hetschko C. (2016). On the misery of losing self-employment. Small Bus. Econ..

[66] Gevaert J., De Moortel D., Wilkens M., Vanroelen C. (2018). What’s up with the self-employed? A cross-national perspective on the self-employed’s work-related mental well-being. SSM-Popul. Health.

[67] Bernhard-Oettel C., Leineweber C., Westerlund H. (2019). Staying in or switching between permanent, temporary and self-employment during 2008–2010: Associations with changing job characteristics and emotional exhaustion. Econ. Ind. Democr..

[68] Sanchez-Garcia J.C., Vargas-Morua G., Hernandez-Sanchez B.R. (2018). Entrepreneurs’ well-being: A bibliometric review. Front. Psychol..

[69] Kamerade D., Wang S.H., Burchell B., Balderson S.U., Coutts A. (2019). A shorter working week for everyone: How much paid work is needed for mental health and well-being?. Soc. Sci. Med..

[70] Taylor J. (2018). Working extra hours in the Australian public service: Organizational drivers and consequences. Rev. Public Pers. Adm..

[71] Seitz J., Rigotti T. (2018). How do differing degrees of working-time autonomy and overtime affect worker well-being? A multilevel approach using data from the German socio-economic panel (soep). Ger. J. Hum. Resour. Manag..

